# Sanguinarine promotes healthspan and innate immunity through a conserved mechanism of ROS-mediated PMK-1/SKN-1 activation

**DOI:** 10.1016/j.isci.2022.103874

**Published:** 2022-02-04

**Authors:** Fang Liu, Haijuan Wang, Xinting Zhu, Nian Jiang, Feng Pan, Changwei Song, Chunbo Yu, Changyan Yu, Ying Qin, Jing Hui, Sanhua Li, Yi Xiao, Yun Liu

**Affiliations:** 1 Guizhou Provincial College-based Key Lab for Tumor Prevention and Treatment with Distinctive Medicines, Zunyi Medical University, Zunyi, GZ 563000, China; 2 College of Basic Medicine, Zunyi Medical University, Zunyi, GZ 563000, China; 3 Institute of Life Sciences, Zunyi Medical University, Zunyi, GZ 563000, China

**Keywords:** Biological sciences, Molecular biology, Immunology

## Abstract

The longevity of an organism is influenced by both genetic and environmental factors. With respect to genetic factors, a significant effort is being made to identify pharmacological agents that extend lifespan by targeting pathways with a defined role in the aging process. Sanguinarine (San) is a benzophenanthridine alkaloid that exerts a broad spectrum of properties. In this study, we utilized *Caenorhabditis elegans* to examine the mechanisms by which sanguinarine influences aging and innate immunity. We find that 0.2 μM sanguinarine extends healthspan in *C. elegans*. We further show that sanguinarine generates reactive oxygen species (ROS), which is followed by the activation of PMK-1/SKN-1pathway to extend healthspan. Intriguingly, sanguinarine increases resistance to pathogens by reducing the bacterial burden in the intestine. In addition, we also find that sanguinarine enhances innate immunity through PMK-1/SKN-1 pathway. Our data suggest that sanguinarine may be a viable candidate for the treatment of age-related disorders.

## Highlights

• Sanguinarine extends healthspan in *C. elegans*
• Sanguinarine-induced ROS activates the PMK-1/SKN-1 pathway to extend healthspan
• Sanguinarine increases resistance to pathogens by reducing the bacterial burden
• Sanguinarine enhances innate immunity through PMK-1/SKN-1 pathway

## Graphical abstract

**Figure:**
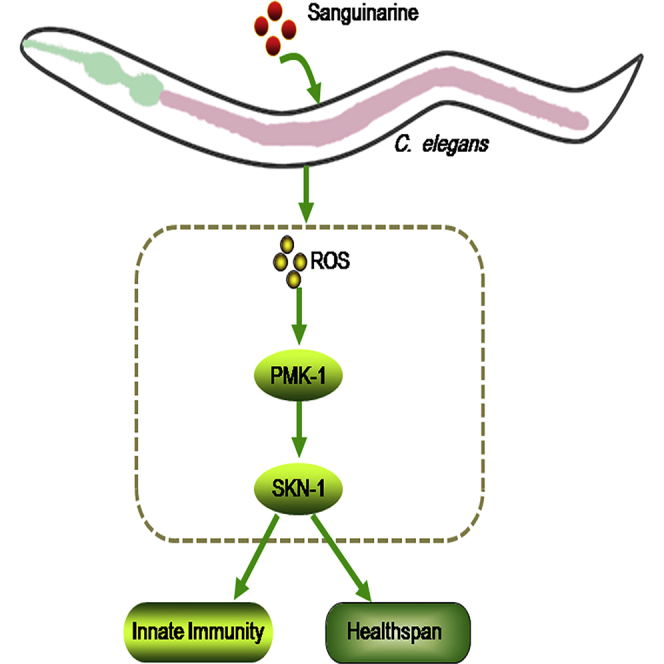

## Introduction

Aging is a multifaceted biological process, and exploration of its mechanisms has attracted attention for a considerable time. Much investigations have been made to discovery of novel anti-aging agents and pharmacological mechanisms of deceleration of the aging process (Zhao et al., 2020). Traditional herbal medicines, containing various natural biologically active compounds, have therapeutic efficacy with minimal adverse effects, providing sources for developing first-line anti-aging drugs (Koehn and Carter, 2005; Zhang et al., 2017). Sanguinarine is a benzophenanthridine alkaloid isolated from the root of Sanguinaria canadensis and other poppy fumaria species (Beaudoin and Facchini, 2014; Ma et al., 2017). Previous studies have found that sanguinarine has broad-spectrum pharmacological properties, including anti-microbial, anti-fungal, anti-tumor, anti-oxidative, and anti-inflammatory effects (Slaninova et al., 2014; Zhang et al., 2018). Whether sanguinarine influences the anti-aging effects remains unknown. However, the molecular mechanisms by which it promotes healthspan and innate immunity have never been examined.

The oxidative stress theory of aging proposes that reactive oxygen species (ROS) generated by normal metabolism cause damage to macromolecules within the cell and that the accumulation of this damage over time leads to cellular dysfunction and eventually organismal death (Harman, 1956, 1972; Sohal and Weindruch, 1996; Van Raamsdonk and Hekimi, 2009). ROS are deemed to reduce the lifespan in *Caenorhabditis elegans* by damaging cellular components (Che,ong et al., 2011; Lee et al., 1999; Vanfleteren and De Vreese, 1996). For example, the mitochondrial complex *mev-1* and *gas-1* mutants exhibit increased ROS levels and shortened mean lifespan (Hartwig et al., 2009; Ishii, 2000; Kayser et al., 2001). However, the traditional view of ROS as a disruptive factor in the aging process has gradually been modified (Back et al., 2012; Scudellari, 2015) and it is now widely accepted that certain levels of ROS are necessary for normal cellular function and can actually promote longevity (Gusarov et al., 2017; Lee et al., 2010; Ristow and Zarse, 2010; Shore and Ruvkun, 2013). Most strikingly, disruption of glucose metabolism increased ROS levels and extended the lifespan of *C. elegans* (Schulz et al., 2007). Sublethal doses of oxidants, or elevated endogenous ROS, were shown to promote the lifespan in *C. elegans* (Lee et al., 2010; Schmeisser et al., 2013c; Van Raamsdonk and Hekimi, 2012; Yang and Hekimi, 2010). In contrast, the antioxidants often fail to change lifespan and may actually accelerate aging (Bjelakovic et al., 2007; Schmeisser et al., 2013c; Yang and Hekimi, 2010). Here, we used *C. elegans* to investigate the mechanism and role of ROS in sanguinarine-mediated healthspan extension.

One major regulator of the stress response is the Nrf protein ortholog SKN-1, a transcription factor that promotes longevity (Hu et al., 2017) and innate immunity by inducing genes involved in detoxification of ROS (Hoeven et al., 2011; Papp et al., 2012; Tang and Pang, 2016). SKN-1 induces phase II detoxification gene transcription. In the intestine, the main detoxification organ in *C. elegans*, SKN-1 accumulates in nuclei and activates target genes that include glutathione-S-transferase 4 (*gst-4*), the g-glutamine cysteine synthetase heavy chain (*gcs-1*), and a wide range of genes that are involved in membrane, lysosomal, and proteasomal homeostasis in response to oxidative stresses, longevity, and innate immunity (Cheong et al., 2011; Hu et al., 2017; Oliveira et al., 2009; Papp et al., 2012). SKN-1 activity is regulated by phosphorylation-dependent control of subcellular localization. The phosphorylation of SKN-1 by specific kinases such as p38 MAPK/PMK-1 can change translocation from the cytosol to the nucleus, where the transcription can then be initiated (Hu et al., 2017; Inoue et al., 2005). Furthermore, ROS could also act as signaling molecules to induce SKN-1 activation in a p38/PMK-1-dependent manner (Hoeven et al., 2011; Inoue et al., 2005; Tang and Pang, 2016). Here, we investigated the role for sanguinarine in healthspan and host defense in *C. elegans*. We found that sanguinarine promoted healthspan and innate immunity by governing ROS and PMK-1/SKN-1 activation. The evolutionary conservation of ROS and PMK-1/SKN-1-dependent manner suggests that the sanguinarine-mediated impact on cellular redox status, healthspan, and innate immunity might be universal.

## Results

### Sanguinarine extends the lifespan and healthspan in *C. elegans*

**Figure 1:**
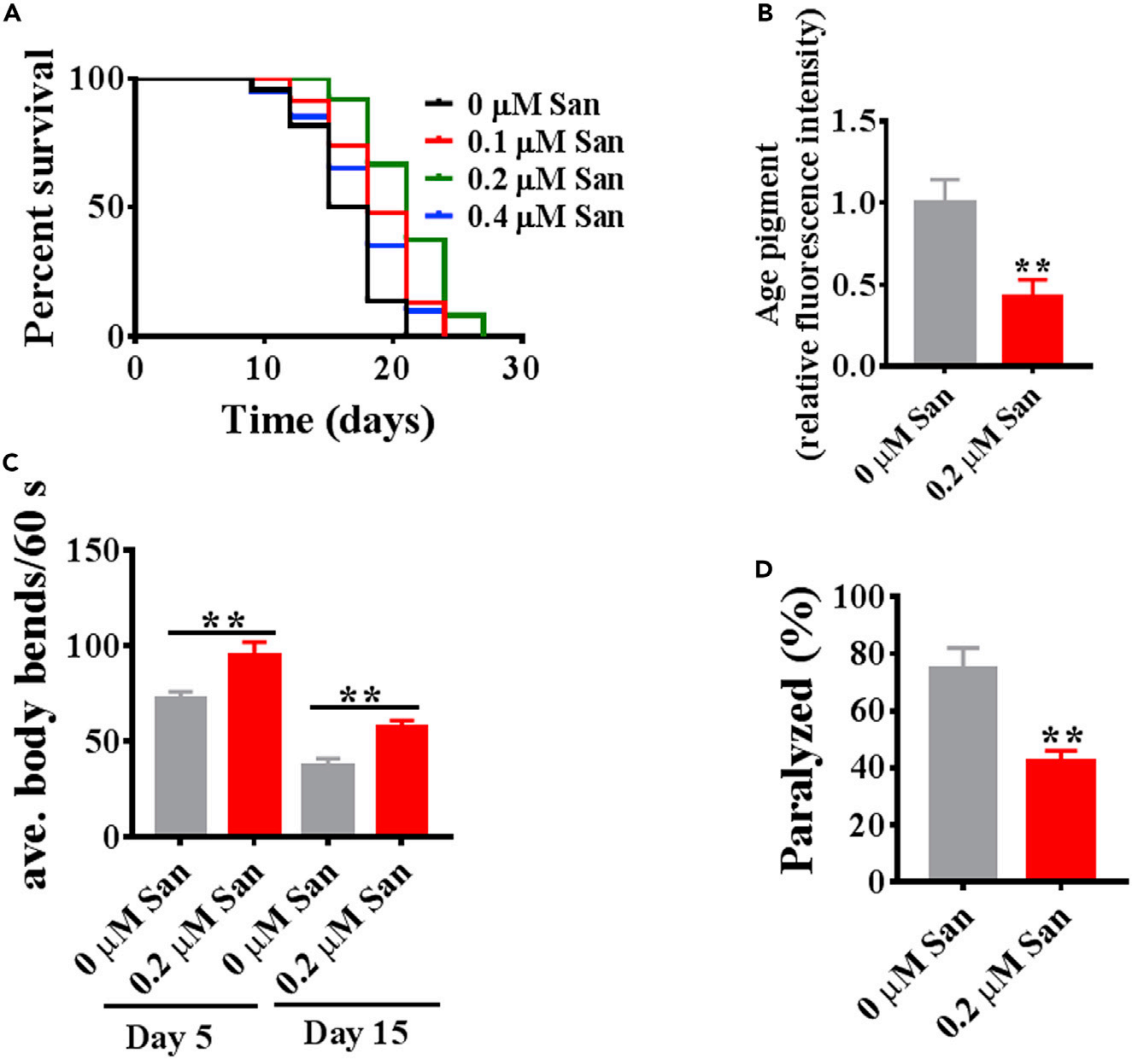
Sanguinarine extends the lifespan and healthspan in *C. elegans* (A)Kaplan-Meier survival curves of N2 hermaphrodite worms exposed to increasing concentrations of San from day 1 of adulthood (p < 0.05; log rank test). (A) See Table S1 for lifespan data. (B) Quantification of “aging pigments” by measuring lipofuscin fluorescence normalized to autofluorescence (n ≥ 20). (∗p < 0.05, unpaired t-test). (C) Locomotion as determined by the number of body bends per min (n ≥ 20). (∗p < 0.05, unpaired t-test). (D) Percentage of paralyzed AM44 mutant worms after treatment with 0.2 μM San (n ≥ 20). (∗p < 0.05, unpaired t-test). Error bars represent mean ± SEM of three independent biological replicates. San is the abbreviation for Sanguinarine.

We tested the effects on worm lifespan of sanguinarine. Sanguinarine at 0.1, 0.2, and 0.4 μM increased mean lifespan by 16%, 25%, and 9% (Figure 1A; Table S1). These results suggest that sanguinarine exhibits a saturating effect on longevity, maximal at 0.2 μM drug, and declining at 0.4 μM drug (Figure 1A; Table S1). As they age, *C. elegans* exhibited muscle deterioration and locomotion rate decline, used as a marker for health and rate of aging in *C*. *elegans* (Chen et al., 2017; Huang et al., 2004; Onken and Driscoll, 2010). 0.2 μM sanguinarine treatment increases the locomotory ability (determined by the average bends of the worm body per 60 s) and decreases age pigments in *C. elegans* (Figures 1B and 1C). The strain AM44 is a mutant model of polyglutamine neurodegenerative diseases of *C. elegans*, in which polyQ proteins are expressed throughout the nematoda nervous system (Rozanov et al., 2020), treatment with 0.2 μM sanguinarine was found to protect from paralysis-inducing effects(Figure 1D). Taken together, these results indicate that sanguinarine not only extends lifespan but also has pronounced health-beneficial effects for the *C*. *elegans*.

### Sanguinarine generates a health-beneficial ROS signal to prolong lifespan through activation *skn-1*

**Figure 2:**
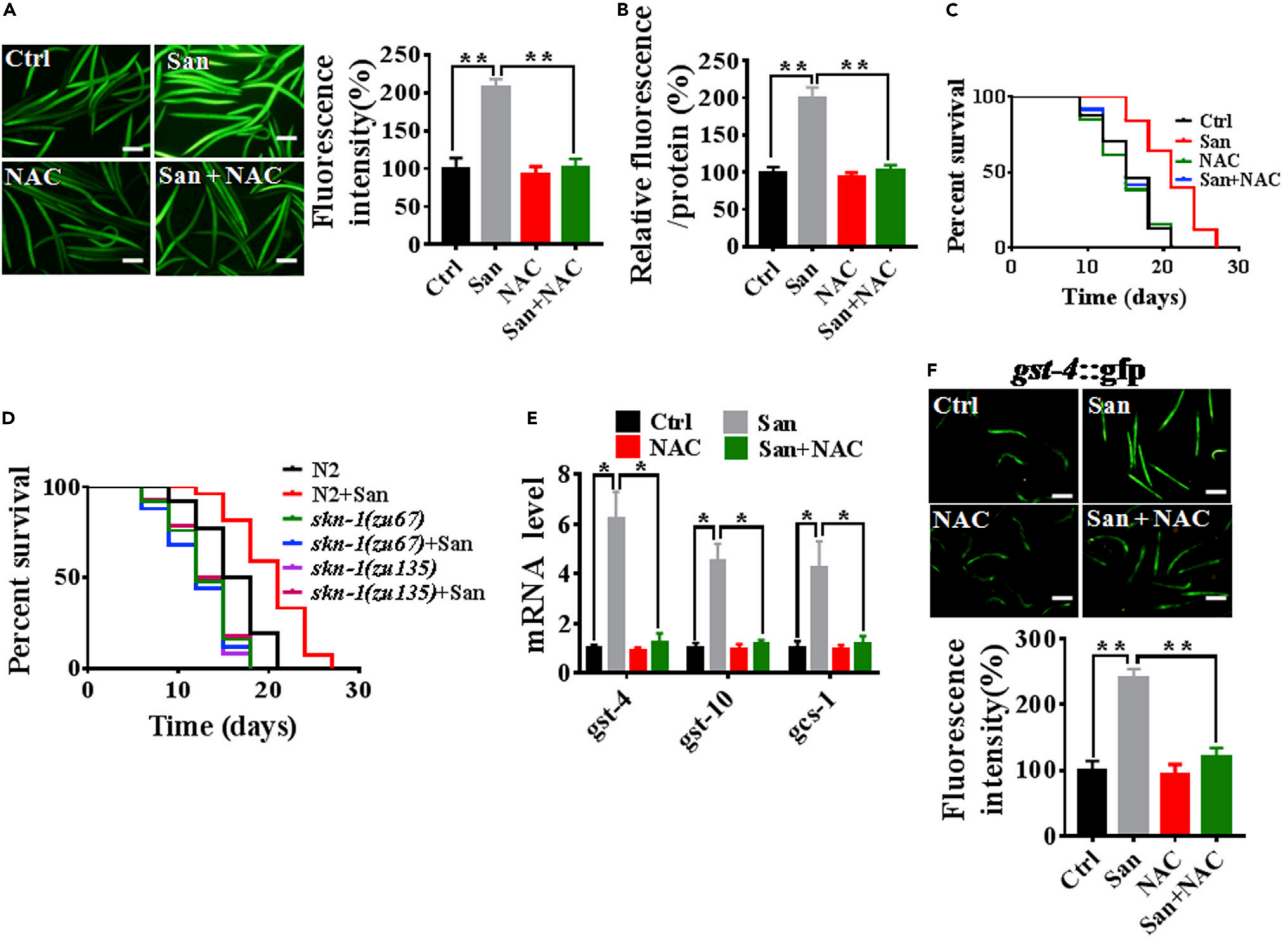
Sanguinarine generates a health-beneficial ROS signal to prolong lifespan through activation *skn-1* (A and B) Quantitation of intracellular levels of ROS in animals treated with 0.2 μM San or NAC and those in untreated controls at day 1 adulthood. Scale bars: 100 μm. San is the abbreviation for Sanguinarine, and NAC represents N-acetyl-cysteine. (∗p < 0.05, unpaired t-test). Error bars represent mean ± SEM of three independent biological replicates. (C) Survival of animals treated with 0.2 μM San or NAC and the untreated controls. (p < 0.05; log rank test). (D) Survival of *skn-1(zu67)* and *skn-1(zu135)* treated with 0.2 μM San and the untreated controls. (log rank test). (C-D) See Table S1 for lifespan data. (E) qPCR analysis of the mRNA level of target genes *skn-1* when worms were exposed to 0.2 μM San or NAC versus the control. (∗p < 0.05, unpaired t-test). Error bars represent mean ± SEM of three independent biological replicates. (F) Images and quantification of GFP fluorescence of transgenic strain *gst-4*p:GFP. Scale bars: 100 μm. (∗p < 0.05, unpaired t-test). Error bars represent mean ± SEM of three independent biological replicate.

Having been proved that sanguinarine promotes the lifespan and healthspan in *C*. *elegans*, we sought to better understand the molecular mechanisms for this observation. Low degrees of metabolic stress, such as ROS generation, have repeatedly been shown to be associated with beneficial outcomes via activation of protective signaling pathways (Cheong et al., 2011; Rozanov et al., 2020; Schmeisser et al., 2013a). The ROS levels were examined by 2′, 7′-dichlorofluorescein diacetate (DCF-DA), a well-known dye sensitive to ROS in *C. elegans* (Tang and Pang, 2016; Zou et al., 2013), and we found that 0.2 μM sanguinarine increased ROS, while treatment with ROS inhibitor N-acetyl-L-cysteine (NAC) completely abolished the effects of sanguinarine on ROS (Figures 2A and 2B). Meanwhile, 0.2 μM sanguinarine extended lifespan, single-treatment NAC did not influence the lifespan, Co-treatment of *C. elegans* with 0.2 μM sanguinarine and 1 mM NAC, showed that NAC significantly reduced the lifespan-extending effect upon sanguinarine (Figure 2C; Table S1). These results suggested that sanguinarine generated a health-beneficial ROS signal to prolong lifespan.

SKN-1, a key transcription factor involved in oxidative stress, longevity, and innate immunity, is activated by ROS (Cheong et al., 2011; Dehghan et al., 2017; Hoeven et al., 2011; Papp et al., 2012; Schmeisser et al., 2013a). We exposed the *skn-1(zu67)* and *skn-1(zu135)* nematode mutants to 0.2 μM sanguinarine and found the lifespan-extending effects of sanguinarine to be abolished (Figure 2D; Table S1). Similar results are also observed in *skn-1* RNAi worms (Figure S1; Table S1). This suggests that the transcription factor *skn-1* is essential for the effects of sanguinarine on lifespan extension. Next, to evaluate the possible function of sanguinarine production ROS on SKN-1 activation, we tested the expression of SKN-1-targeted genes, *gst-4*, *gst-10*, and *gcs-1* (Cheong et al., 2011). Quantitative real-time PCR analysis demonstrated that SKN-1-dependent genes were upregulated in sanguinarine-treated animals compared with control (Figure 2E). However, NAC reduced their expression in the sanguinarine-treated worms (Figure 2E). Furthermore, we detected the expression of *gst-4* through using the transgenic worms expressing *gst-4p::GFP*. We observed higher levels of GFP in sanguinarine-treated animals, but NAC abolished the GFP levels of sanguinarine-treated nematodes (Figure 2F). To further characterize SKN-1 activation, we tested SKN-1::GFP nuclear translocation. We found that sanguinarine significantly increased SKN-1 nuclear accumulation in the intestine (Figure S2). Overall, these data indicated that sanguinarine promoted ROS generation to extend lifespan via the *skn-1* activation.

### Sanguinarine generates a health-beneficial ROS signal to prolong lifespan dependent on *pmk-1*/*skn-1* activation

**Figure 3:**
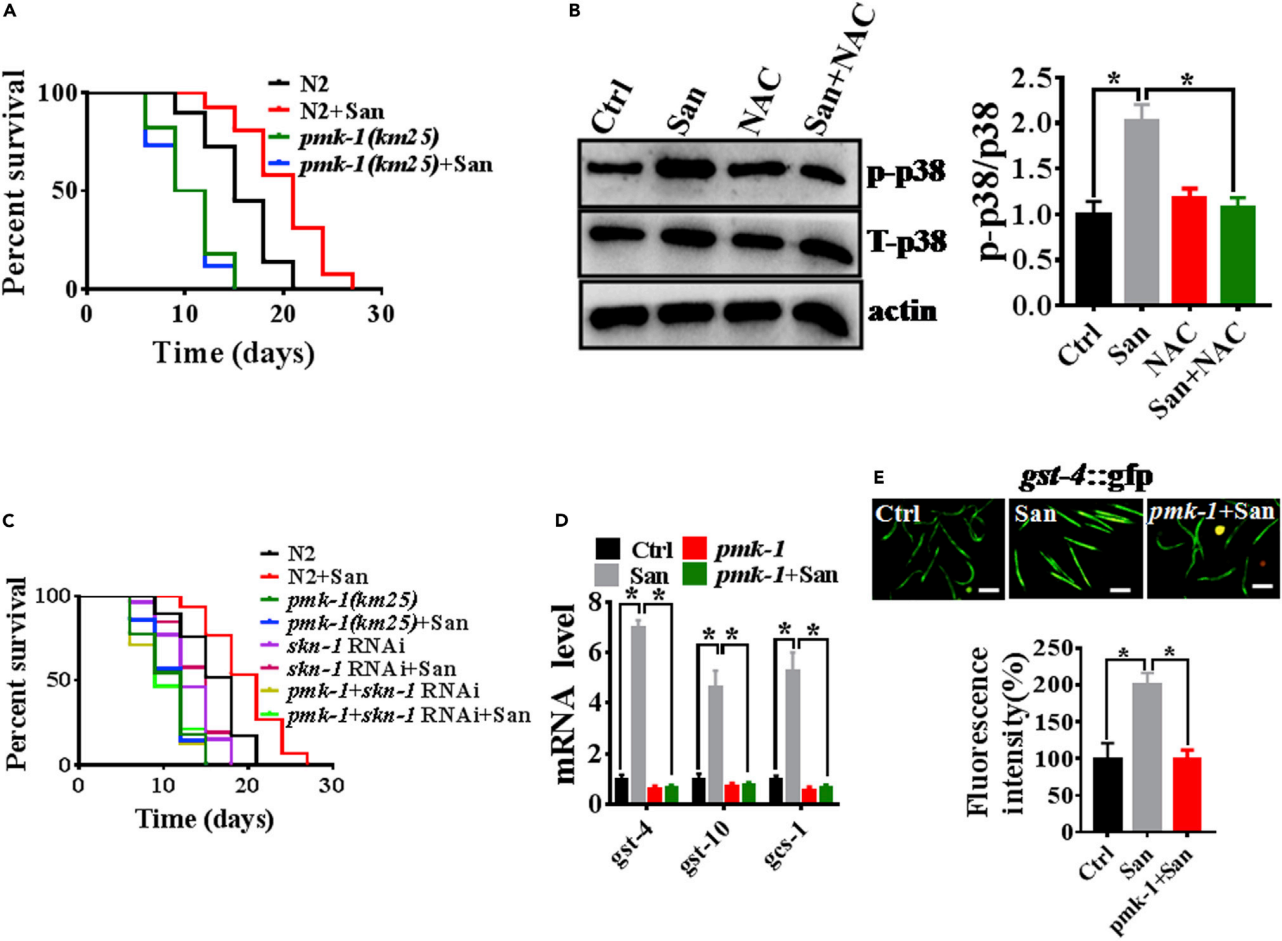
PMK-1/p38 MAPK mediated sanguinarine generation ROS-induced SKN-1 activation on lifespan of *C. elegans* (A) Survival of *pmk-1(km25)* treated with 0.2 μM San and the untreated controls. (log rank test). (A) See Table S1 for lifespan data. (B) Western blot analysis of the phosphorylated p38 MAPK levels when worms were exposed to 0.2 μM San or NAC versus the control. (∗p < 0.05, unpaired t-test). Error bars represent mean ± SEM of three independent biological replicates. (C) Survival of *pmk-1(km25)*, *skn-1*RNAi, and *pmk-1(km25)*;*skn-1*RNAi treated with 0.2 μM San and the untreated controls. (log rank test). (C) See Table S1 for lifespan data. (D) qPCR analysis of the mRNA level of target genes *skn-1* in 0.2 μM San-treated worms or *pmk-1(km25)* mutants versus the control. (∗p < 0.05, unpaired t-test). Error bars represent mean ± SEM of three independent biological replicates. (E) Images and quantification of GFP fluorescence of transgenic strain *gst-4*p:GFP in 0.2 μM San-treated worms or *pmk-1(km25)* mutants versus the control. Scale bars: 100 μm. (∗p < 0.05, unpaired t-test). Error bars represent mean ± SEM of three independent biological replicates.

Previous studies had shown that PMK-1/p38 MAPK, mediated ROS-induced SKN-1 activation under oxidative and pathogenic stresses (Hoeven et al., 2011; Inoue et al., 2005). Therefore, sanguinarine generated ROS to prolong lifespan in *C. elegans* via PMK-1/p38 MAPK pathway. We found that 0.2 μM sanguinarine failed to extend lifespan in *pmk-1(km25)* mutants, compared with WT worms (Figure 3A; Table S1). These results indicated that sanguinarine promoted lifespan dependent on PMK-1/p38 MAPK. To determine whether sanguinarine activated p38 MAPK signaling or not, we tested the level of phosphorylation PMK-1, which is a signature of its activation (Ren et al., 2009; Sun et al., 2011). We found that 0.2 μM sanguinarine increased the phosphorylation levels of p38 MAPK in *C.elegans* (Figure 3B). In contrast, NAC significantly reduced the protein levels of active PMK-1 in sanguinarine-treated *C. elegans* (Figure 3B). These results indicated that sanguinarine governed ROS production and subsequent PMK-1/p38 MAPK activation. Therefore, the ROS induced by sanguinarine activated the SKN-1 via p38 MAPK/PMK-1 on lifespan of *C. elegans*. We found that 0.2 μM sanguinarine failed to extend lifespan in *pmk-1(km25)* mutants, *skn-1* RNAi worms, and *pmk-1(km25);skn-1* RNAi worms, compared with WT worms (Figure 3C; Table S1). In addition, we tested the expression of SKN-1-targeted genes, *gst-4*, *gst-10*, and *gcs-1* (Cheong et al., 2011). Quantitative real-time PCR analysis demonstrated that SKN-1-dependent genes were upregulated in sanguinarine-treated animals compared with control (Figure 3D). *pmk-1(km25)* mutant worms decreased their expression. However, sanguinarine failed to increase their expression in the *pmk-1(km25)*worms (Figure 3D). Furthermore, we detected the expression of *gst-4* through using the transgenic worms expressing *gst-4p::GFP*. We observed higher levels of GFP in sanguinarine-treated animals, but not in *pmk-1(km25)* worms (Figure 3E). Overall, these data suggested that sanguinarine promoted ROS-induced SKN-1 activation to extend lifespan via the PMK-1/p38 MAPK pathway.

### Sanguinarine enhances innate immunity in *C. elegans*

**Figure 4:**
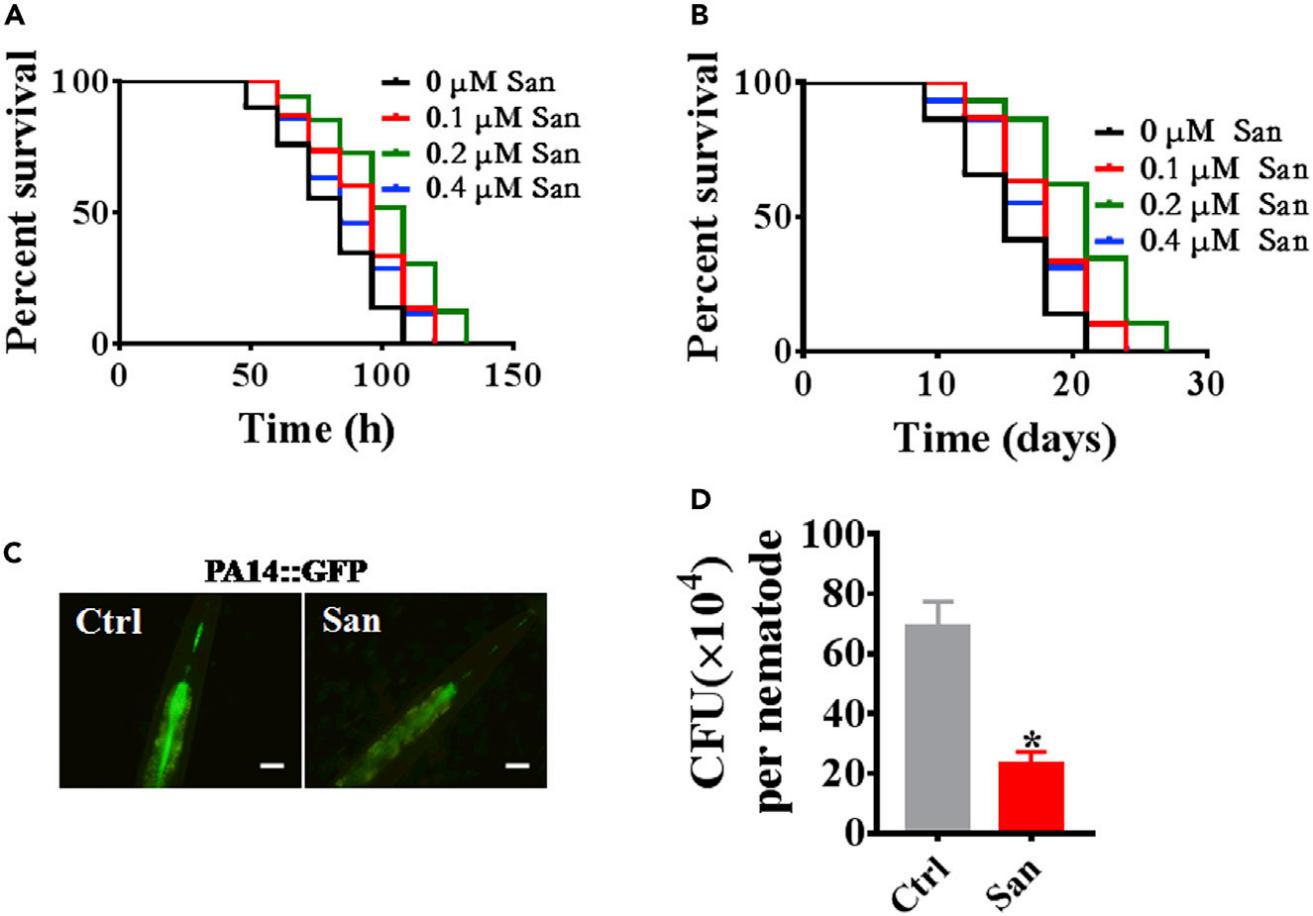
Sanguinarine enhances innate immunity in *C. elegans* (A) Survival of N2 hermaphrodite worms exposed to increasing concentrations of San in response to *P. aeruginosa* PA14 infection. (p < 0.05; log rank test). (B) Survival of N2 hermaphrodite worms exposed to increasing concentrations of San in response to heat-killed *P. aeruginosa* PA14 infection. (p < 0.05; log rank test). (A–B) See Table S2 for survival data. (C) WT animals treated with 0.2 μM San were exposed to *P. aeruginosa* expressing GFP for 48 h and then visualized using a Zeiss Axioskop two plus fluorescence microscope. Scale bars: 50 μm. (D) WT animals treated with 0.2 μM San were exposed to *P. aeruginosa* expressing GFP for 48 h and the colony-forming units (CFU) were quantified (n ≥ 10). (∗p < 0.05, unpaired t-test). Error bars represent mean ± SEM of three independent biological replicates.

To investigate whether sanguinarine enhances the innate immunity, worms were exposed to the human opportunistic pathogen *Pseudomonas aeruginosa*(PA14), and we found that wild-type animals treated with sanguinarine (0.1, 0.2, and 0.4 μM) exhibited increased resistance to *P. aeruginosa* in dose-dependent manner (Figure 4A; Table S2). Like lifespan, sanguinarine exhibited a saturating effect on pathogen resistance, maximal at 0.2 μM drug, and declining at 0.4 μM drug(Figure 4A; Table S2). After 0.2 μM sanguinarine treatment, nematodes exposed to *Staphylococcus aureus*, *Enterococcus faecalis*, or *Salmonella enterica* also increased survival rate (Figures S3A, 3B, and 3C; Table S2). These results indicated that sanguinarine promoted the innate immunity in *C. elegans*. Meanwhile, to understand whether sanguinarine affects the expression of *irg-1p::GFP* (Dunbar et al., 2012) and *T24B8.5p::GFP*(Shivers et al., 2009), which are induced by *P. aeruginosa*, sanguinarine increased the expression of *irg-1p::GFP* and *T24B8.5p::GFP*(Figures S4A and 4B). In addition, like feeding *Escherichia coli* OP50, we observed that sanguinarine (0.1, 0.2, and 0.4 μM) also extended the lifespan of wild-type (WT) animals that were fed heat-killed *P. aeruginosa* PA14 (Figure 4B; Table S1). The clearance of the bacterial load is part of host defense against pathogen infection (Sun et al., 2011). Compared to control animals, sanguinarine-treated animals reduced the accumulation pattern of *P. aeruginosa*/green fluorescent protein (GFP) (Figure 4C). Furthermore, the number of bacterial cells in sanguinarine-treated worms was decreased in the *C. elegans* intestine to that in control animals (Figure 4D). Overall, these results suggested that sanguinarine reduced the bacterial burden in the *C. elegans* intestine to enhance the pathogens resistance.

### Sanguinarine promotes pathogens resistance via *pmk-1*/*skn-1*-dependent manner

**Figure 5:**
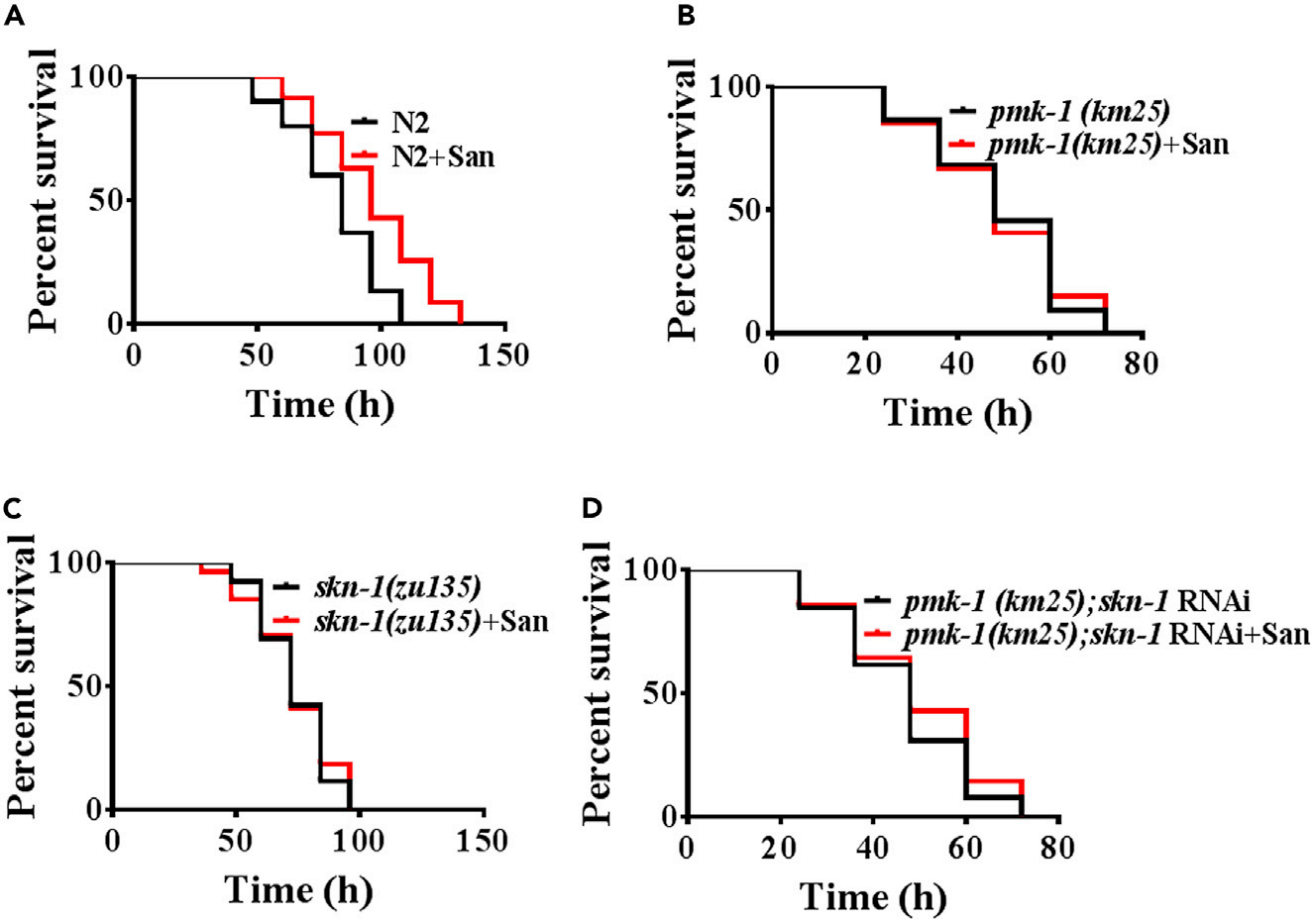
Sanguinarine promotes pathogens resistance via *pmk-1*/*skn-1*-dependent manner (A) Survival of N2 hermaphrodite worms exposed to increasing concentrations of San in response to *P. aeruginosa* PA14 infection. (p < 0.05; log rank test). (B) Survival of *pmk-1(km25)* treated with 0.2 μM San and the untreated controls in response to *P. aeruginosa* PA14 infection. (log rank test). (C) Survival of *skn-1(zu135)* treated with 0.2 μM San and the untreated controls in response to *P. aeruginosa* PA14 infection. (log rank test). (D) Survival of *pmk-1(km25)*;*skn-1* RNAi treated with 0.2 μM San and the untreated controls in response to *P. aeruginosa* PA14 infection. (log rank test). (A-D) See Table S2 for survival data.

The PMK-1(p38 MAPK)/SKN-1-dependent manner had an important role in pathogen resistance (Papp et al., 2012). Like lifespan, sanguinarine confers protection against pathogens via PMK-1/SKN-1-dependent manner. After 0.2 μM sanguinarine treatment, worms exposed to *P. aeruginosa* (PA14) increased survival rate (Figure 5A; Table S2). However, we found that 0.2 μM sanguinarine failed to enhance resistance to *P. aeruginosa* PA14 infection in *pmk-1(km25)*, *skn-1(zu135)* mutants, and *pmk-1(km25);skn-1* RNAi worms, compared with WT worms (Figures 5B–5D; Table S2). ROS are produced by mitochondria and are involved in basic immune pathways that directly kill pathogens (Tang and Pang, 2016). To determine whether sanguinarine governs activation of mitochondrial unfolded protein response (UPR^mt^) or not, we use UPR^mt^ reporter strains *hsp-6p::GFP* in *c. elegans* (Pellegrino et al., 2014). We found that sanguinarine did not increase the *hsp-6p::GFP* activation in the intestine (Figure S5A). ATFS-1, a transcription factor, which mediates the UPR^mt^ to pathogens resistance (Pellegrino et al., 2014), sanguinarine enhanced the resistance to *P. aeruginosa* in *atfs-1* RNAi worms (Figure S5B). These results suggested that sanguinarine did not influence the mitochondrial unfolded protein response. Taken together, these results suggested that sanguinarine acted on the PMK-1/SKN-1 pathway to promote innate immunity in *C. elegans*.

## Discussion

Aging is also at least partially caused by oxidative stress. According to Harman's “free-radical theory of aging”, aging results from damage caused by ROS that accumulate over time, leading to cellular dysfunction and an increased probability of death (Van Raamsdonk and Hekimi, 2012). In this regard, ROS can be classified as toxic agents. Recently, however, several studies have shown that ROS damage is not causally involved in the aging process but that ROS levels are correlated with the aged phenotype because ROS as signaling molecules alleviate the cellular stresses caused by aging (Hekimi et al., 2011; Yee et al., 2014). On the other hand, ROS are required to regulate cell proliferation, to sustain the cellular redox homeostasis, and to induce the activation of specific signaling pathways as intracellular messenger molecules (Kampkotter et al., 2007).

Most strikingly, previous studies have shown that moderate mitochondrial dysfunction (Felkai et al., 1999; Feng et al., 2001; Yang and Hekimi, 2010), severe loss of mtROS detoxification (Van Raamsdonk and Hekimi, 2009), and increased mtROS generation (Yang and Hekimi, 2010), hormesis effects (Bora et al., 2021; Yee et al., 2014), as well as treatments with pro-oxidants (Heidler et al., 2010; Lee et al., 2010; Van Raamsdonk and Hekimi, 2012; Yang and Hekimi, 2010), can all prolong lifespan (Yee et al., 2014). However, the traditional view of ROS as a disruptive factor in the aging process has gradually been modified (Back et al., 2012; Scudellari, 2015) and it is now widely accepted that certain levels of ROS are necessary for normal cellular function and can actually promote longevity (Gusarov et al., 2017; Lee et al., 2010; Ristow and Zarse, 2010; Shore and Ruvkun, 2013). Sanguinarine, a benzophenanthridine alkaloid, induces apoptosis in human colorectal cancer HCT-116 cells through ROS-mediated Egr-1 activation and mitochondrial dysfunction (Han et al., 2013). We find that sanguinarine generates a health-beneficial ROS signal to prolong lifespan. Our work provides additional support for the notion that sanguinarine exerts their lifespan-promoting effects by transiently producing a ROS signal that constitutively induces PMK-1/SKN-1 activation.

In this study, we have shown that sanguinarine extends healthspan and innate immunity. We reveal that 0.2 μM sanguinarine treatment increases the locomotory ability and decreases age pigments in *C. elegans*, which used as a marker for health and rate of aging in *C*. *elegans*. In addition, 0.2 μM sanguinarine was found to protect from paralysis-inducing effects. Taken together, these results indicate that sanguinarine not only extends lifespan but also has pronounced health-beneficial effects for the *C*. *elegans*. Consistent with previous work, the ROS signal initiates PMK-1/SKN-1-mediated redox response to increase stress resistance and fitness that finally culminates in lifespan extension (Schmeisser et al., 2013c). We find that sanguinarine generates a health-beneficial ROS signal to promote lifespan dependent on *pmk-1*/*skn-1* activation. Intriguingly, sanguinarine enhances the innate immunity in *C. elegans* to response the Gram-negative pathogens *P. aeruginosa* and *S. enterica* as well as the Gram-positive pathogens *Enterococcusfaecalis* and *S. aureus* via PMK-1/SKN-1-dependent manner. Considering its mechanism of action extensive evolutionary conservation, our results suggest that sanguinarine might also have a role in mammalian aging and innate immunity.

### Limitations of the study

We have shown that sanguinarine promotes healthspan and enhances innate immunity in *C. elegans* through activation PMK-1/SKN-1-dependent manner. However, it remains to be determined whether sanguinarine in mammals also influence healthspan and innate immunity. ROS are produced by mitochondria and are involved in basic immune pathways that directly kill pathogens. Our work suggests that sanguinarine does not influence the mitochondrial unfolded protein response. Future work should investigate how sanguinarine treatment leads to ROS production.

## STAR★Methods

### Key resources table

**Table:** 

REAGENT or RESOURCE	SOURCE	IDENTIFIER
**Antibodies**		

Rabbit polyclonal anti-active p38	Abcam	Cat# ab4822
Rabbit anti-p38 antibody	Abcam	Cat# ab170099
Rabbit anti-beta actin	Abcam	Cat# ab227387

**Bacterial and virus strains**		

*E.coli* OP50	Yun Nan University	N/A
*S. enterica* SL1344	Yun Nan University	N/A
*E. faecalis*	ATCC	ATCC 29212
*P. aeruginosa* PA14	Yun Nan University	N/A
*S. aureus* NCTC8325	Yun Nan University	N/A

**Chemicals, peptides, and recombinant proteins**		

DCF-DA	Solarbio	Cat# D6470
NAC	Solarbio	Cat# IA0050
TRIzol Reagent	Invitrogen	Cat#15596026
IPTG	Sigma-Aldrich	CAS:367-91-1

**Experimental models: Organisms/strains**		

EU1 *skn-1(zu67)*IV/nT1[unc-?(n754) let-?](IV;V)	Caenorhabditis Genetics Center	WB Strain: EU1
EU31 *skn-1(zu135)* IV/nT1[unc-?(n754) let-?]	Caenorhabditis Genetics Center	WB Strain: EU31
KU25 *pmk-1(km25)*	Caenorhabditis Genetics Center	WB Strain: KU25
CL2166[*gst-4p::gfp*]	Caenorhabditis Genetics Center	WB Strain: CL2166
SJ4100[*hsp-6p::gfp*]	Caenorhabditis Genetics Center	WB Strain: SJ4100
AU133[*irg-1p::gfp*]	Caenorhabditis Genetics Center	WB Strain: AU133
LD1[*skn-1::gfp*]	Caenorhabditis Genetics Center	WB Strain:LD1
AU78[*T24B8.5p::gfp*]	Caenorhabditis Genetics Center	WB Strain: AU78

**Software and algorithms**		

GraphPad Prism	GraphPad	https://www.graphpad.com/scientific-software/prism/ RRID: SCR_002798
ImageJ	Schneider et al., 2012	https://imagej.nih.gov/ij/

### Resource availability

#### Lead contact

Further information and requests for resources and reagents should be directed to and will be fulfilled by the Lead Contact, Yun Liu (liuyunzmu@126.com).

#### Materials availability

All data generated or analyzed in this study are included in this published article.

### Experimental model and subject details

Experiments were performed in compliance with the guidelines of Guizhou Provincial College-based Key Lab for Tumor Prevention and Treatment with Distinctive Medicines of Zunyi Medical University.

#### Nematode strains and maintenance

Worms were maintained and propagated nematode growth medium (NGM) agar-containing plates seeded with OP50 *E. coli* bacteria at 20°C (Brenner, 1974; Stiernagle, 2006). The following nematodes strains were obtained from the Caenorhabditis Genetics Center (CGC), which is funded by NIH Office of Research Infrastructure Programs (P40 OD010440): N2 Bristol wild-type, EU1 *skn-1(zu67)*IV/nT1[unc-?(n754) let-?](IV;V), EU31 *skn-1(zu135)* IV/nT1[unc-?(n754) let-?], KU25 *pmk-1(km25)*, CL2166[*gst-4p::gfp*], AM44, SJ4100[*hsp-6p::gfp*], AU133[*irg-1p::gfp*], AU78[*T24B8.5p::gfp*], LD1[*skn-1::gfp*]. *C. elegans* mutants were backcrossed three times into the WT strain (N2) and used in the laboratory.

### Method details

#### RNA interference

The strains of *E.coli* used for RNAi were obtained from the Ahringer library. (Kamath and Ahringer, 2003). RNAi feeding experiments were performed on synchronized L1 to L2 larvae at 20°C. Briefly, *E. coli* strain HT115(DE3) expressing dsRNA was grown overnight in LB broth containing 100 μg/ml ampicillin at 37°C, and then spread to NGM plates containing 100 μg/ml ampicillin and 5 mM isopropyl 1-thio-β-D-galactopyranoside (IPTG). The RNAi-expressing bacteria were grown overnight at 25°C. Synchronized L1 to L2 larvae were placed on RNAi plates until they reach maturity at 20°C. *Unc-22* RNAi was included as a positive control in all experiments to account for RNAi efficiency.

#### Infection assay

*E.coli* OP50, *S. enterica* SL1344, *E. faecalis* ATCC 29212, and *P. aeruginosa* PA14 were grown overnight in LB broth at 37°C, and *S. aureus* NCTC8325 was grown overnight in tryptic soy broth (TSB, BD, Sparks, MD) at 37°C, were then spread to NGM plates. All infection assay were performed on NGM agar plates or NGM plates supplemented with or without sanguinarine (0, 0.1, 0.2, 0.4 μM). Synchronized populations of worms were cultivated on *E.coli* OP50 at 20°C until the young adult stage (i.e., within 12 h beyond the L4 stage) (without sanguinarine). Next, 50-60 worms were transferred to NGM agar plates supplemented with or without sanguinarine (0, 0.1, 0.2, 0.4 μM) and containing *S. enterica* SL1344, *S. aureus* NCTC8325, and *P. aeruginosa* PA14 at 25°C, respectively for infection assay . The number of living worms was counted at 12 h intervals. Immobile adult worms unresponsive to touch were scored as dead. Three plates were tested per assay and all experiments were performed three times independently.

#### Fluorescence microscopy

Synchronized L1 worms of the *gst-4*::GFP strain were cultivated on *E.coli* OP50 at 20°C until the young adult stage (i.e., within 12 h beyond the L4 stage), next 100 worms were transferred to agar plates supplemented with or without 0.2 μM sanguinarine or 1 mM NAC at 12h. The images were obtained using a Zeiss Axioskop 2 plus fluorescence microscope (Carl Zeiss, Jena, Germany) with a digital camera. Fluorescence intensity was quantified by using the Image J software (NIH). Three plates of about 40 animals per plate were tested per assay and all experiments were performed three times independently.

#### Lifespan assays

All lifespan assays were performed at 20 °C according to standard protocols (Schulz et al., 2007). Briefly, synchronized L1 to L2 larvae were placed on NGM plates until they reached maturity at 20°C. After L4 around 120 nematodes were manually transferred to fresh incubation plates containing the sanguinarine (0, 0.1, 0.2, 0.4 μM). N-acetyl cysteine (NAC) was dissolved in water (for NAC, 500-fold stock solution, 500 mM). For the first 10–14 days, worms were transferred every day and afterwards every second day. Nematodes that show no reaction to gentle stimulation were scored as death. Those animals that crawled off the plates or displayed non-natural death particularly due to internal hatching were censored (Schmeisser et al., 2013b). Three plates (d=3 cm) of 50-80 worms per plates were tested per assay and all experiments were performed three times independently

#### Locomotion assays

##### Nematodes were synchronized and treated with or without 0.2 μM sanguinarine

Nematodes were synchronized and treated with or without 0.2 μM sanguinarine starting at L4 larvae stage. On days 5 and 15 of life, 30 individuals from the control and experimental plates were measured for body bend rate in liquid. Briefly, worms were placed in 20 μl M9 buffer on a glass slide and filmed with a Zeiss Imager M2 microscope. Body bends were counted by reviewing each frame of the 60 s film(Onken and Driscoll, 2010).

##### Age pigment fluorescence detection

Nematodes were synchronized and treated for 10 days with or without 0.2 μM sanguinarine starting at L4 larvae stage. Worms were mounted onto an agarose pad attached to a glass slide and photographed with Zeiss Imager M2 microscope. Fluorescence intensity was measured by Image J(Chen et al., 2017).

##### Measurement of ROS

The ROS levels were detected by 2, 7-dichlorodihydrofluorescein diacetate (DCF-DA) as a probe (Tang and Pang, 2016; Zou et al., 2013). Briefly, after treated with 0.2 μM sanguinarine or 1 mM NAC for 24 h, about 1200 worms from each group were collected in M9 buffer and washed three times to remove bacteria. Then, worms were homogenized in PBST (PBS with 1% Tween 20) and centrifuged at 12,000 rpm at 4 °C. 100 μL supernatants were mixed with equal volume 100 mM DCF-DA prepared in PBS at 37°C. Fluorescent intensity was measured by Spectra Max M5 fluorescent microplate reader (Molecular Devices, Sunnyvale, CA) at the excitation wavelength 488 nm and emission wavelength 535 nm after 1 hr incubation. Samples were read kinetically every 20 min for 2.5 h. The protein content in supernatant was determined by bicin-choninic acid (BCA) assay to normalize the fluorescent intensity.

##### Quantitative real-time PCR

Nematodes were synchronized and treated for 1 day with or without 0.2 μM sanguinarine starting at L4 larvae stage. Total RNA was extracted from worms with TRIzol Reagent (Invitrogen) (Lapierre et al., 2013). Random-primed cDNAs were generated by reverse transcription of the total RNA samples with SuperScript II (Invitrogen) and qPCR analysis was conducted using SYBR Premix-Ex TagTM (Takara, Dalian, China) on an Applied Biosystems Prism 7000 Sequence Detection System (Applied Biosystems, Foster City, CA). Using *pmp-3* for an internal control as previously described. (Lapierre et al., 2013). The following primers were used for this study:

*pmp-3* primers:

*pmp-3*-F: TGGATTGTCATTGGCGTCG

*pmp-3*-R: GTTGTCGCAGAGTGGTGTTT

*gst-4* primers:

*gst-4*-F: TCGGTCAGTCAATGTCTATCAC

*gst-4*-R: CGGAAAAAGAATATGAAATCTCTGTAT

*gst-10* primers:

*gst-10*-F: ATGCTCCTTGGTCAGTTGCC

*gst-10*-R: TTGCTCGTTGGATCCGTTC

*gcs-1* primers:

*gcs-1*-F: CAGGTGAATGCGATGCTTGG

*gcs-1*-R: CAAGCGATGAGACCTCCGTA

##### Quantification of intestinal bacterial loads

Synchronized populations of worms were cultivated on *E.coli* OP50 at 20°C until the young adult stage. *P. aeruginosa*/GFP were grown in LB liquid medium containing ampicillin (100 μg/ml) at 37°C overnight and plated onto NGM plates. Worms then were transferred to NGM agar plates (supplemented with or without 0.2 μM of sanguinarine) containing *P. aeruginosa*/GFP for 48h at 25°C (Sun et al., 2011). To eliminate the *P. aeruginosa*/GFP around the surface of worms, worms were transferred to NGM agar plate seeded with *E. coli* OP50 for 15 min for three times (Sun et al., 2011). Ten worms were transferred into 50 microliters PBS plus 0.1% Triton and ground (Sun et al., 2011). The lysates were serially diluted by 10-folds in sterilized water and spread onto LB agar plates/ampicillin at 37°C. After one day of incubation at 37°C, colonies of *P. aeruginosa* /GFP were counted. Five plates were tested per assay and all experiments were performed three times independently.

##### Western blotting

Nematodes were synchronized and treated for 1 day with or without 0.2 μM sanguinarine starting at L4 larvae stage. After worms were homogenized in liquid nitrogen, the homogenate was lysed on ice for 60 minutes in lysis buffer (BioTeKe). The lysates of total protein were loaded (40μg per well) and separated on a 10% SDS polyacrylamide gel. Proteins were then transferred to immobilon-PSQ transfer PVDF membrane (Millipore, Bedford, MA). Phosphorylated PMK-1 protein was detected using an anti-active p38 polyclonal antibody from rabbit (1:1000 dilution; Abcam, ab4822), anti-p38 antibody from rabbit (1:1000 dilution; Abcam, ab170099) and anti-beta actin antibody (1:1000 dilution; Abcam, ab227387). The secondary antibody was a peroxidase-coupled anti-rabbit IgG (1:20000 dilution; Abmart). Blots were developed using Super Signal chemiluminescence substrate (Pierce). Band intensities were measured using Image J software.

### Quantification and statistical analysis

Data were presented as mean ± SEM. Statistical analyses for all data except for lifespan assays was carried out using Student's t-test (unpaired, two-tailed) or ANOVA after testing for equal distribution of the data and equal variances within the data set. Survival data were analyzed by using the log-rank (Mantel-Cox) test. p < 0.05 was considered significant. Data were analyzed using the SPSS17.0 software (IBM, Armonk, New York).

## Data Availability

• Data reported in this paper will be shared by the lead contact upon request. • This paper does not report original code. • Any additional information required to reanalyze the data reported in this paper is available from the lead contact upon request.

## Supplemental information

Document S1. Figures S1–S5

Table S1. The lifespan data related to Figures 1, 2, 3, 4B, and S1

Table S2. The survival data related to Figures 1, 2, 3, 4B, S3, and S5B
