# Supplementary material for: Sanguinarine promotes healthspan and innate immunity through a conserved mechanism of ROS-mediated PMK-1/SKN-1 activation
Source: iScience. 2022 Feb 4;25(3):103874. doi: 10.1016/j.isci.2022.103874 (PMC8857505; doi:10.1016/j.isci.2022.103874)

iScience, Volume *25*

Supplemental information

Sanguinarine promotes healthspan and innate immunity through a conserved mechanism of ROS-mediated PMK-1/SKN-1 activation

Fang Liu, Haijuan Wang, Xinting Zhu, Nian Jiang, Feng Pan, Changwei Song, Chunbo Yu, Changyan Yu, Ying Qin, Jing Hui, Sanhua Li, Yi Xiao, and Yun Liu

Figure S1 Sanguinarine extends lifespan through *skn-1*, related to Figure 2.

Survival of *skn-1*RNAi treated with 0.2 μM San and the untreated controls. (P<0.05;log-rank test). EV(empty vector). See Table S1 for lifespan data.

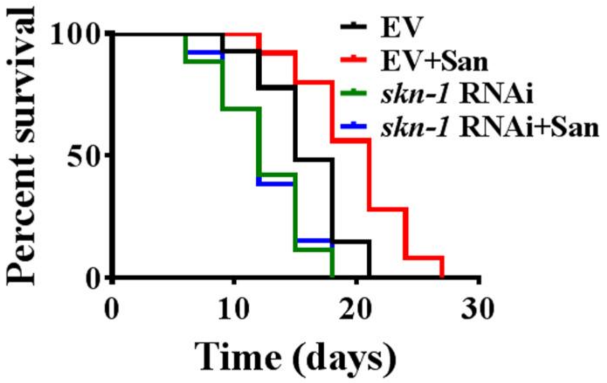

Figure S2 The localization changes of SKN-1 by sanguinarine, related to Figure 2.

(n ≥10). (*P<0.05, Chi-squared test). Scale bars: 50 μm. These results performed 3 independent biological replicates.

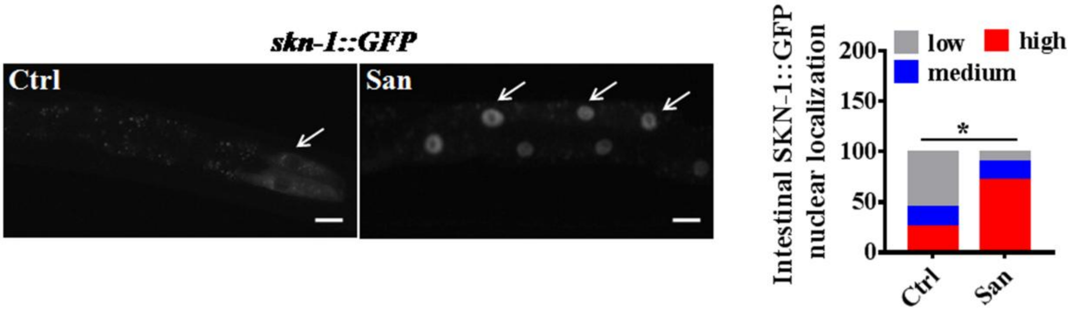

Figure S3 Sanguinarine enhance the resistance to *Staphylococcus aureus*, *Enterococcus faecalis*, or *Salmonella enterica*, related to Figure 4.

Survival of N2 hermaphrodite worms treated with 0.2 μM San and the untreated controls in response to *Staphylococcus aureus* (A), *Enterococcus faecalis* (B), or *Salmonella enterica* (C). (P<0.05;log-rank test). (A–C) See Table S2 for survival data.

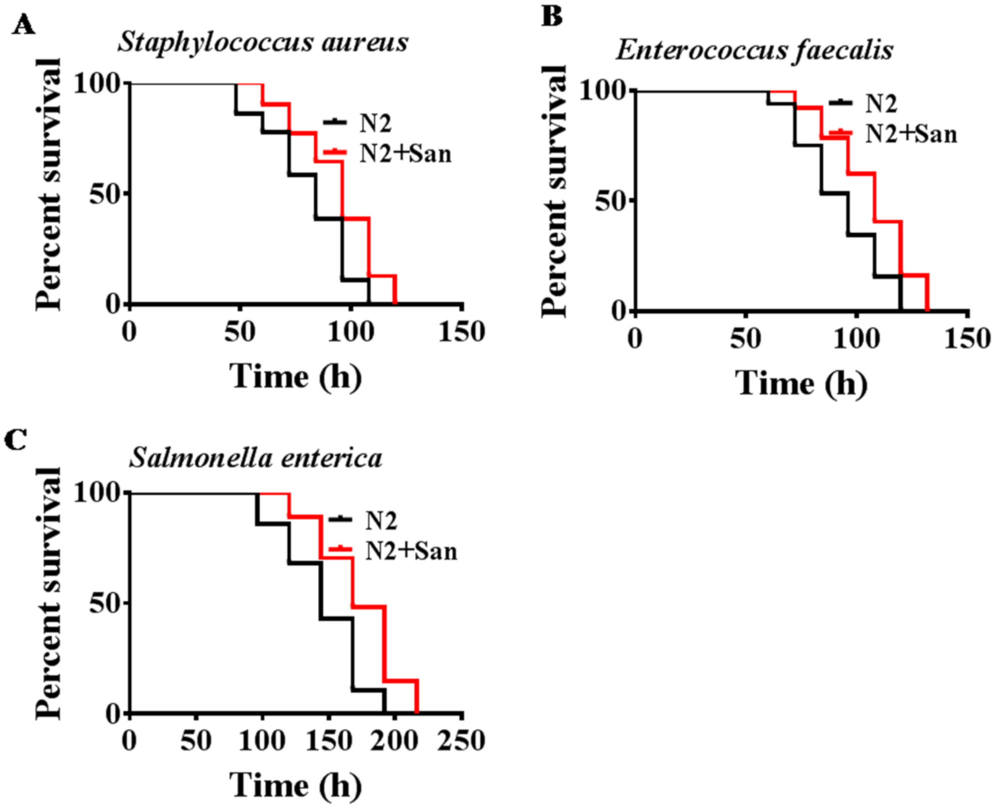

Figure S4 The expression changes of *irg-1::GFP* and *T24B8.5::GFP* by sanguinarine, related to Figure 4. sanguinarine increased the expression of *irg-1p::GFP* (A) and *T24B8.5p::GFP* (B). Scale bars: 100 μm. (n ≥10) (*P< 0.05, unpaired t-test). Error bars represent mean ± SEM of 3 independent biological replicates.

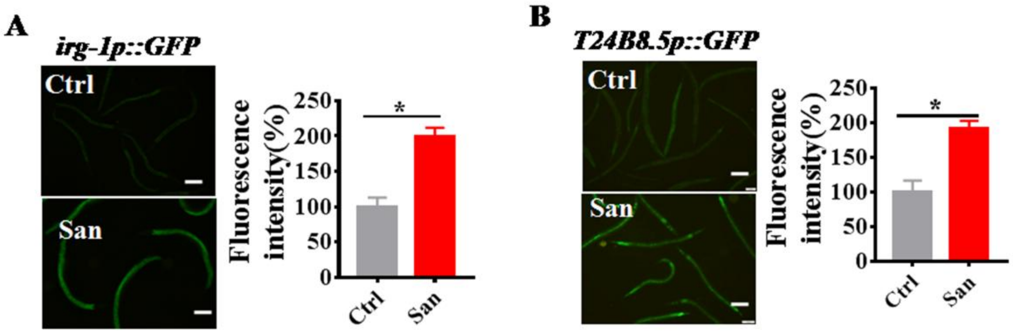

Figure S5 sanguinarine did not influence the mitochondrial unfolded protein response, related to Figure 5. sanguinarine did not increase the *hsp-6p::GFP* activation in the intestine(n ≥10) (A). (P=0.148, unpaired t-test). Scale bars: 100 µm. Error bars represent mean ± SEM of 3 independent biological replicates. NS (no significance) sanguinarine enhanced the resistance to *P. aeruginosa* in *atfs-1* RNAi worms (B). (P<0.05; log-rank test). (B) See Table S2 for survival data.

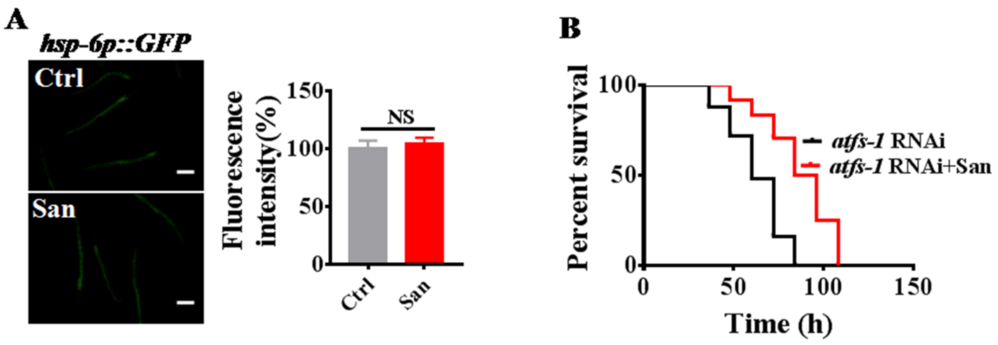

Supplement: Document S1. Figures S1–S5 [file mmc1.pdf]
